# The Value of Diffusion Kurtosis Imaging in Detecting Delayed Brain Development of Premature Infants

**DOI:** 10.3389/fneur.2021.789254

**Published:** 2021-12-13

**Authors:** Xin Zhao, Chunxiang Zhang, Bohao Zhang, Jiayue Yan, Kaiyu Wang, Zitao Zhu, Xiaoan Zhang

**Affiliations:** ^1^Department of Imaging, Third Affiliated Hospital of Zhengzhou University, Zhengzhou, China; ^2^McGill University, Montreal, QC, Canada; ^3^MRI Research, GE Healthcare, Beijing, China; ^4^Wuhan University, Wuhan, China

**Keywords:** brain, neurodevelopment, preterm infants, MRI, diffusion kurtosis imaging

## Abstract

**Objective:** Preterm infants are at high risk of the adverse neurodevelopmental outcomes. Our aim is to explore the value of diffusion kurtosis imaging (DKI) in diagnosing brain developmental disorders in premature infants.

**Materials and Methods:** A total of 52 subjects were included in this study, including 26 premature infants as the preterm group, and 26 full-term infants as the control group. Routine MRI and DKI examinations were performed. Mean kurtosis (MK), radial kurtosis (RK), fractional anisotropy (FA), and mean diffusivity (MD) values were measured in the brain regions including posterior limbs of the internal capsule (PLIC), anterior limb of internal capsule (ALIC), parietal white matter (PWM), frontal white matter (FWM), thalamus (TH), caudate nucleus (CN), and genu of the corpus callosum (GCC). The chi-squared test, *t*-test, Spearman's correlation analysis, and receiver operating characteristic curve were used for data analyses.

**Results:** In the premature infant group, the MK and RK values of PLIA, ALIC, and PWM were lower than those in the control group (*p* < 0.05). The FA values of PWM, FWM, and TH were also lower than those of the control group (*p* < 0.05). The area under curves of MK in PLIC and ALIC, MD in PWM, and FA in FWM were 0.813, 0.802, 0.842, and 0.867 (*p* < 0.05). In the thalamus and CN, the correlations between MK, RK values, and postmenstrual age (PMA) were higher than those between FA, MD values, and PMA.

**Conclusion:** Diffusion kurtosis imaging can be used as an effective tool in detecting brain developmental disorders in premature infants.

## Introduction

In the recent years, the birth rate of premature babies has increased significantly in many countries (1). At the same time, with the development of monitoring and treatment technology in the neonatal intensive care unit (NICU), the survival rate of premature babies has also been greatly improved. Compared with full-term babies, the brain developments of premature babies are impaired due to the young gestational age (GA) (2). To provide objective indicators for clinical evaluation of brain development status, it would be necessary to quantitatively analyze the brain development of preterm and full-term infants.

Traditional diffusion tensor imaging (DTI) technique has been used to study the brain development of premature infants. Although pathological specimens can directly show the degree of brain development, these cannot reflect the level of brain development in a living state. DTI technology can non-invasively provide quantitative parameters to reflect the development of white matter in the living body, with the integrity of tissue evaluated from the microscopic field and the white matter (WM) fibers and fiber bundles of the brain tissue being observed (3). Many studies have shown that the more mature the development of neonatal WM, the higher the fractional anisotropy (FA) value and the lower the diffusion coefficient apparent diffusion coefficient (ADC) value (4, 5). When applying diffusion-weighted imaging (DWI) and DTI to assess WM development, the theoretical premise of these two models is that the diffusion of water molecules is normally distributed (6). However, the diffusion distribution of human brain water molecules is dominated by non-normal distribution (7, 8). The current DWI and DTI evaluation methods still have certain limitations.

Diffusion kurtosis imaging (DKI) is an extension of DTI technology, which describes the degree of water diffusion deviating from the normal distribution in tissues. The kurtosis information reflects the non-Gaussian characteristics caused by the complex structure. It better reflects the changes in the microstructure of brain gray matter and WM (9), thus, be more suitable for grasping the microstructure changes. In the recent years, DKI has been used to evaluate brain development. The DKI has detected significant microstructural changes consistent with known patterns of brain maturation (10). In addition, study has shown that DKI shows potential advantages in detecting normal brain development in children (11), but few studies have focused on brain development in premature babies, which may help understand the early neurodevelopmental characteristics of premature babies. In this study, we aimed to discuss the value and advantages of DKI in evaluating the brain development of preterm infants with multiple parameters.

## Materials and Methods

### Subjects

A total of 52 newborns underwent MRI examination from January 2020 to May 2021, including 26 premature infants and 26 full-term infants. Before the examination, the doctor informed the guardian of the purpose and potential risks and obtained informed parental consent. This study was approved by the Ethics Committee.

All the recruited infants met the following inclusion criteria: no chromosomes or major congenital diseases; no intracranial infection, sepsis and other infectious diseases; no hypoglycemic encephalopathy, bilirubin encephalopathy, and other encephalopathy. Imaging criteria: routine MRI showed normal results; no obvious motion artifacts. Groups were divided according to the following criteria. GA ≥ 37 weeks (mean GA: 38.67 ± 1.18 weeks) was considered as the term infant group (*n* = 26), and scanned at 40.57 ± 3.07 weeks; GA < 37 weeks (mean GA: 34.22 ± 2.08 weeks) was the premature infant group (*n* = 26), and scanned at term equivalent age [mean postmenstrual age (PMA): 41.53 ± 2.22 weeks].

### Magnetic Resonance Acquisition and Image Analysis

Each newborn was given an intravenous injection of 5 mg/kg of phenobarbital 30 min before the MRI scan. After the newborn fell asleep, he was escorted to the MRI room by the attending physician and his family. The nurse put the swaddled baby on the MRI scan bed, then used sponges to properly fix both the sides of the head. Finally, antinoise earplugs were placed in the external auditory canal to reduce the impact of MRI equipment noise.

All the MRI scan were carried out on 3.0 T MR scanner (Pioneer, GE Healthcare, Milwaukee, Wisconsin, USA) with T1WI flair, T2WI flair, DWI, and DKI (TR = 2,000 ms, TE = 2.32 ms, directions = 30, *b*-value = 0, 1,000, 2,000 mm^2^/s).

The DKI parametric maps were calculated by using the iQuant software (GE Healthcare, Beijing, China). The iQuant is based on the commercial version of software Horos (https://horosproject.org/). Since the newest AW4.7 version cannot support the old platform for DKI processing, the GE development team moved the algorithms including the DKI images processing from AW4.6 FuncTool platform (GE Healthcare, Beijing) to the Horos and named as iQuant. The DKI algorithm was used as plugin in iQuant. Then, the two radiologists of physicists outline 7 regions of interest (ROI) including posterior limbs of the internal capsule (PLIC), anterior limb of internal capsule (ALIC), genu of the corpus callosum (GCC), parietal white matter (PWM), frontal white matter (FWM), thalamus (TH), and lenticular nucleus (LN) which were outlined manually. The ROIs were measured three times bilaterally and an average value was calculated to minimize the error value.

### Statistical Analysis

The statistical analysis was performed on IBM SPSS Statistics 21.0 (IBM Corporation, Armonk, NY, USA). The Student's *t*-test or chi-squared test was used to compare the differences in clinical characteristics between groups. The receiver operating characteristic curve (ROC) was used to analyze the differences among different ROIs. Spearman's correlation analysis was used to analyze the correlation between DKI parameters and PMA. *p* < 0.05 indicated statistical significance.

## Results

### General Demographics of Infants

There was no difference between the preterm group (11 males, 15 females) and term born (14 males, 12 females) in gender, delivery method, mean PMA at MRI, mean birth weight, and mean body weight at MRI (*p* > 0.05), as shown in Table 1. The DKI parameters showed good interobserver agreement, as shown in Table 2.

**Table 1:** General demographics of infants.

	**Preterm infants**	**Term infants**
	**(*n =* 26)**	**(*n* = 26)**
Male%	11 (42.3)	14 (53.8)
Cesarean delivery%	7 (27)	10 (38.5)
Mean GA (SD; week)	34.22 ± 2.08^*^	38.67 ± 1.18
Mean PMA at MRI (SD; week)	40.57 ± 3.07	41.53 ± 2.22
Mean birth weight (SD; kg)	2.66 ± 0.74	3.01 ± 0.31
Mean body weight at MRI (SD; kg)	3.55 ± 0.56	3.68 ± 0.63

*There were no differences between preterm infants group (n = 26) and term born (n = 26) in gender, delivery method, mean PMA at MRI, mean birth weight and mean body weight at MRI(P > 0.05). Mean GA in the preterm groups was lower than that in the term control group (P < 0.05). GA, gestational age; PMA, postmenstrual age*.

* *indicates P < 0.05*.

**Table 2:** Inter-observer consistency of measurements.

**Parameters**	**Intraclass correlation coefficient, 95% CI**
MK	0.894 (0.635–0.993)
RK	0.880 (0.620–0.983)
FA	0.955 (0.716–0.994)
MD	0.903 (0.636–0.993)

*95% CI = 95% confidence interval. MK, mean kurtosis; RK, radial kurtosis; FA, fractional anisotropy; MD, mean diffusivity*.

### Comparison of DKI Parameters Between Preterm Infants and Term Infants

**Figure 1:**
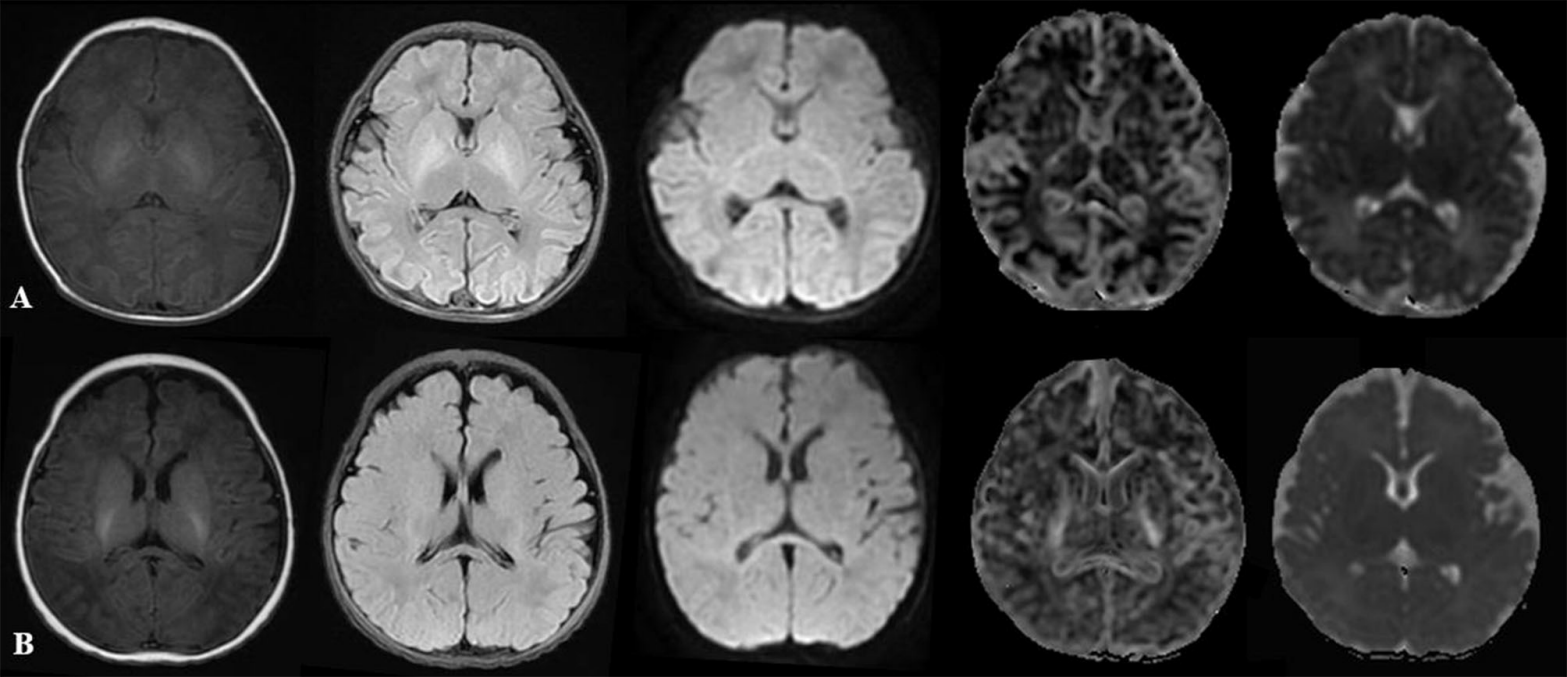
Comparison of multiparameter images in different PMAs. **(A)** represented: male, GA 30 weeks, PMA 42 weeks. **(B)** represented: male, GA 37 weeks, PMA 43 weeks. From left to right: T1WI flair, T2WI flair, DWI, MK, and MD maps. DWI, diffusion-weighted imaging; PMA, postmenstrual age; GA, gestational age; MK, mean kurtosis; MD, mean diffusivity.

Figure 1 displayed T1WI flair, T_2_WI flair, DWI, MK, and MD images of two newborns.

**Figure 2:**
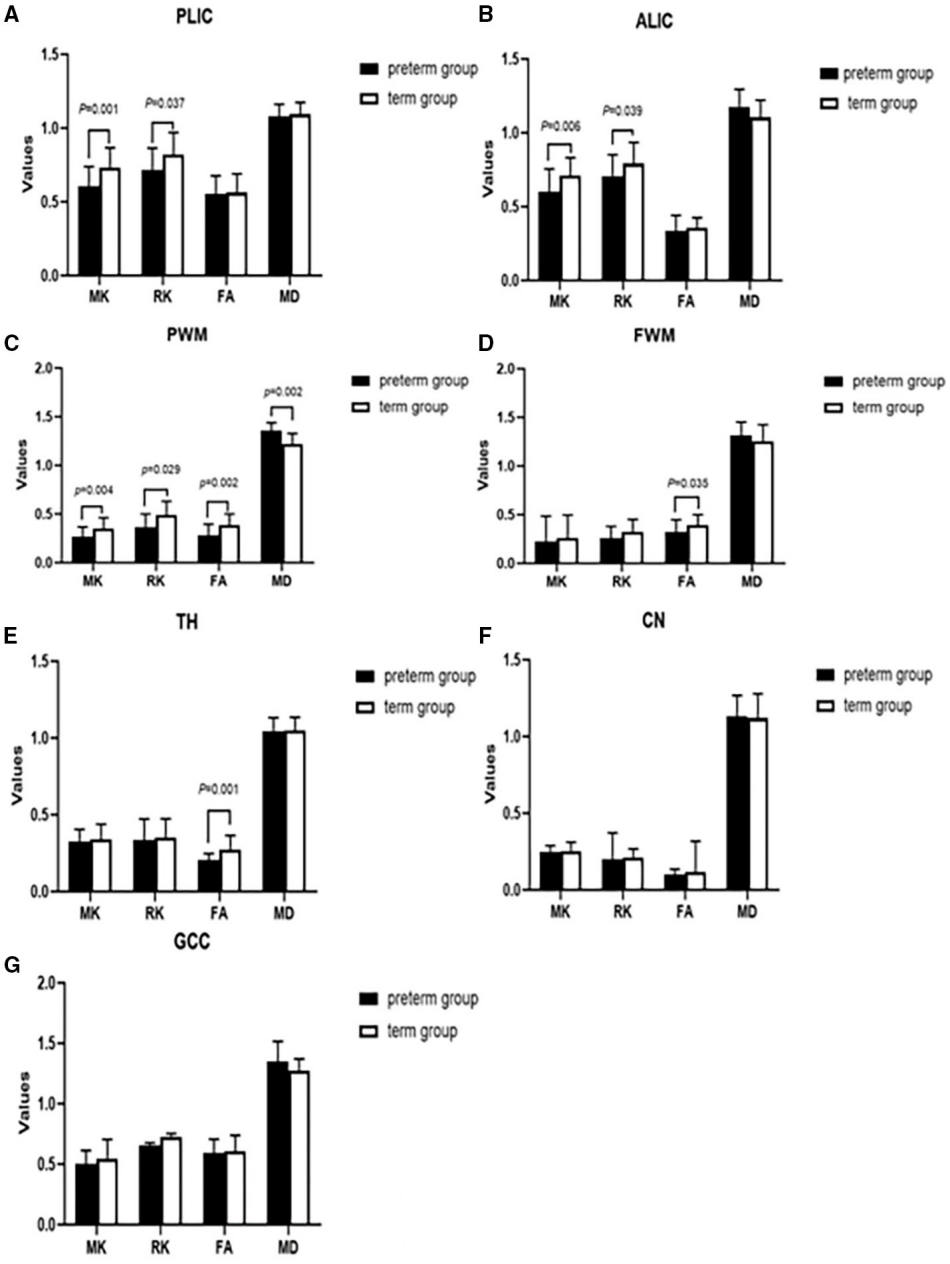
Comparing the difference of parameter values between preterm infants (*n* = 26) and term infants (*n* = 26) in the different brain regions **(A–G)**. Black represented premature infants, white represented full-term infants. PLIC, posterior limbs of the internal capsule; ALIC, anterior limb of internal capsule; PWM, parietal white matter; FWM, frontal white matter; TH, thalamus; CN, caudate nucleus; GCC, genu of the corpus callosum; MK, mean kurtosis; RK, radial kurtosis; FA, fractional anisotropy; MD, mean diffusivity.

As shown in Figure 2, significant differences were found between preterm infants and term infants. In the preterm infant group, the MK value (0.605 ± 0.134 vs. 0.734 ± 0.133, *p* = 0.001) and RK value (0.716 ± 0.148 vs. 0.821 ± 0.150, *p* = 0.037) of PLIC were significantly different from the term infant group. The MK and RK values of ALIC in the term infant group were significantly higher than those of the preterm infant group (0.601 ± 0.154 vs. 0.711 ± 0.121, *p* = 0.006; 0.708 ± 0.144 vs. 0.793 ± 0.142, *p* = 0.039). MK, RK, FA, and MD value were all significantly different from that of the term infant group in PWM (0.268 ± 0.100 vs. 0.355 ± 0.108, *p* = 0.004; 0.366 ± 0.134 vs. 0.489 ± 0.142, *p* = 0.029; 0.282 ± 0.114 vs. 0.386 ± 0.115, *p* = 0.002; 1.357 ± 0.083 × 10^−3^ mm^2^/s vs. 1.221 ± 0.106 × 10^−3^ mm^2^/s, *p* = 0.002, respectively). The FA values of FWM and TH were significantly lower than those of the term infant group (0.320 ± 0.129 vs. 0.392 ± 0.108, *p* = 0.035; 0.205 ± 0.043 vs. 0.275 ± 0.090, *p* = 0.001, respectively).

### Diagnostic Performance of DKI Parameters

By comparing the DKI parameters between the two groups in different regions, the region and parameter with significant differences were obtained. We performed ROC analyses on these regions and parameters. The area under curves (AUC) of MK in PLIC and ALIC were 0.813 and 0.802 with the sensitivity of 75.4, 77.7% and the specificity of 86.9, 80.8% (critical point: 0.703 and 0.649, respectively, *p* < 0.05). The AUC of MD in PWM was 0.842 with a sensitivity of 72.3% and a specificity of 87.8% (critical point: 1.419, *p* < 0.05). Similarly, the AUC of FA in FWM was 0.867 with a sensitivity of 78.5% and a specificity of 92.3% (critical point: 0.469, *p* < 0.05). Details are shown in Table 3.

**Table 3:** Diagnostic performance of DKI parameters in different ROIs.

**ROI**	**Parameters**	**Specificity**	**Sensitivity**	**Critical point**	**AUC**	***P-*value**
PLIC	MK	86.90%	75.40%	0.703	0.813	<0.05^*^
	RK	80.80%	46.20%	0.882	0.638	>0.05
ALIC	MK	80.80%	77.70%	0.649	0.802	<0.05^*^
	RK	69.20%	60.40%	0.793	0.614	>0.05
PWM	MK	63.10%	55.40%	0.344	0.542	>0.05
	RK	73.10%	53.80%	0.42	0.571	>0.05
	FA	52.20%	35.70%	0.727	0.522	>0.05
	MD	87.80%	72.30%	1.419	0.842	<0.05^*^
FWM	FA	92.30%	78.50%	0.469	0.867	<0.05^*^
TH	FA	82.30%	11.50%	0.479	0.665	>0.05

*Diagnostic manifestations of DKI parameters in different ROIs. The AUC of MK in PLIC and ALIC were 0.813 and 0.802 (P < 0.05). The AUC of MD in PWM was 0.842 (P < 0.05). The AUC of FA in FWM was 0.867 (P < 0.05). PLIC, posterior limbs of the internal capsule; ALIC, anterior limb of internal capsule; PWM, parietal white matter; FWM, frontal white matter; TH, thalamus; MK, mean kurtosis; RK, radial kurtosis; FA, fractional anisotropy; MD, mean diffusivity; AUC, area under curves*.

* *indicates P < 0.05*.

### Correlation Between DKI Parameters and PMA in the Gray Matter Areas

**Figure 3:**
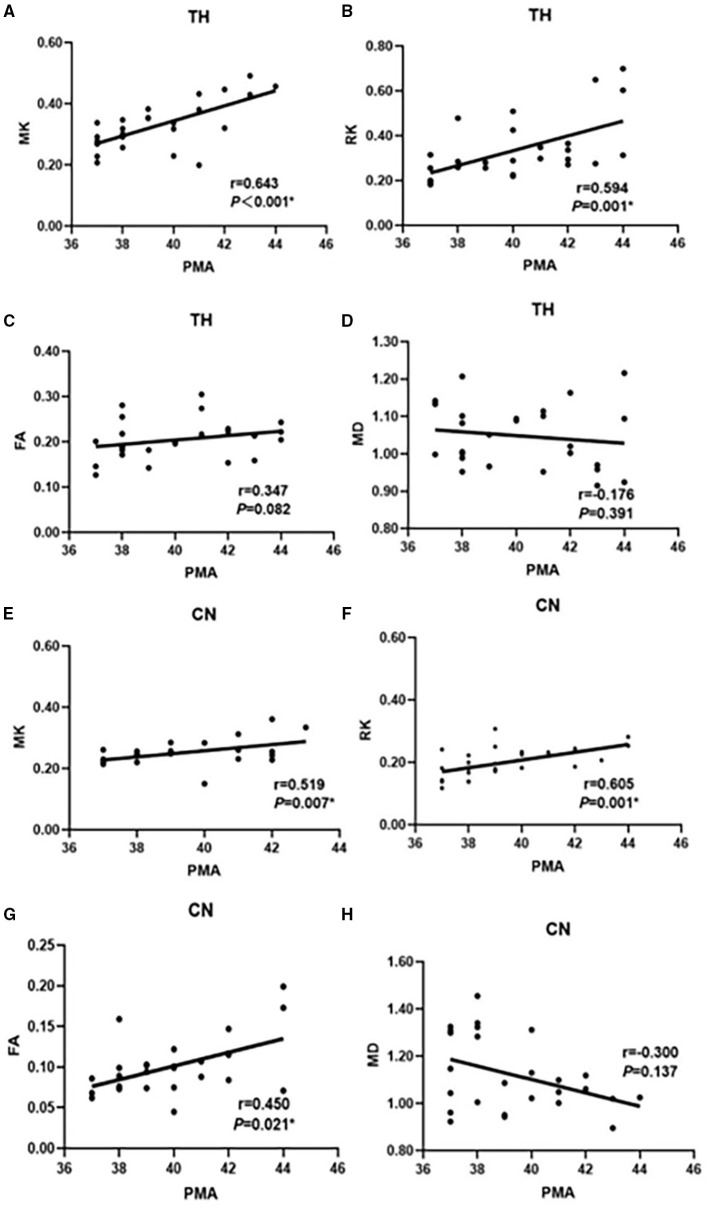
Correlation between MK, RK, FA, MD, and PMA in TH **(A–D)**; Correlation between MK, RK, FA, MD, and PMA in CN **(E–H)** in the preterm infants (*n* = 26). MK and FA regression lines with correlational coefficient (*r*). correlation significance (*P*). The selected ROI are the thalamus and the caudate nucleus. PMA, postmenstrual age; TH, thalamus; CN, caudate nucleus; MK, mean kurtosis; RK, radial kurtosis; FA, fractional anisotropy; MD, mean diffusivity.

As shown in Figure 3, in the thalamus the correlation between MK, RK values, and PMA (*r* = 0.643 and 0.594, respectively, *p* < 0.05) was higher than the correlation between FA, MD values and PMA (*r* = 0.347 and −0.176, respectively, *p* > 0.05). Similarly, the correlation between MK, RK values, and PMA (*r* = 0.519 and 0.605, *p* < 0.05) was greater than the correlation between FA and MD values and PMA (*r* = 0.450, *p* < 0.05; *r* = −0.300, *p* > 0.05) in caudate nucleus (CN).

## Discussion

During the brain development of premature infants, DKI can reveal the microstructure changes and maturation processes in different brain regions. Through comparison with term infants, this study shows that DKI is helpful in diagnosing brain retardation in premature infants. Compared with the parameter FA, the parameters MK and RK of DKI can better capture microstructure changes. When describing gray matter structure, such as TH and CN, MK is better than FA. These results indicate that MK is superior to FA in diagnosing premature infants with delayed brain development. It is more advantageous in discovering changes in the microstructure of the brain.

Because premature birth may lead to relatively slow brain development in premature infants, there are some brain regions that are less developed than the full-term infants. In this study, we found that the MK and RK values of PLIC and ALIC were lower than those of the full-term infants. The fibers that made up the internal capsule (IC) came from the descending fibers of the cerebral cortex. The following factors affected the development of the complex structure of IC (12, 13), i.e., the increase of nerve cell axon diameter, change of nerve cell membrane composition, *myelination of axons continued to improve*, increase in the number of microglia, and decreased extracellular space. The parameters of DKI, such as MK, RK can sensitively reflect these microstructure complexity. This is also in line with Paydar's view that MK and RK allow more comprehensive characterization of the microstructural changes during brain development of the children, especially in PLIC and ALIC (10). Frontal-parietal white matter (FWM, PWM) reflected the degree of brain development in the front and middle brain. In this study, the FA values of PWM and FWM were lower than those in the term infant group. FWM was the language and motor center (14), so it is speculated that the language and motor development of preterm infants is delayed than that of full-term infants. Similarly, when the PWM of premature babies was underdeveloped, the ability to the sense of touch and pain and language was also delayed. The thalamus was the gray matter nucleus, one of the areas where neonatal metabolism was vigorous. Its myelin development was also very active. At the same time, the thalamus was also the highest sensory center and the most important sensory conduction relay station (15–17). The results showed that the FA value of TH was lower than that in the term infants. It was assumed that premature babies were delayed in perception, cognition, and motor development. DKI can be used as an effective tool to assess brain developmental delay in the premature infants.

By comparing DKI parameters in the different brain regions between the two groups, we selected four ROIs with significant differences, including ALIC, PLIC, PWM, and FWM. Our results showed that the AUC of MK in ALIC and PLIC were statistically significant. Studies have shown that the neonatal brain development follows a backward-to-forward pattern. Due to the early myelination of ALIC and PLIC, the metabolism is vigorous and the oxygen demand is high. When exposed to risk factors for preterm birth, the metabolically active areas are the first to be damaged (18). At the same time, MK can reflect the backward development of PLIC and ALIC by capturing the changes in microstructure (19). When brain development is impaired, the decreased density of cells and axon membranes may also lead to decreased MK values, which are significantly different from those in the control group (16). In our study, FA did not show good diagnostic value in ALIC and PLIC. Because the IC acts as a WM plate connecting the upper and lower fibers of the cerebral cortex to the brainstem, the structure is more complicated (20). For this complex structure, the diffusion of water molecules actually deviates from the normal distribution, and MK can quantify this deviation.

Radial kurtosis represents the degree to which the molecular diffusion deviates radially from the Gaussian pattern. Compared with the FA value, RK changes more significantly in the premature brain of the infants (21), which is confirmed in this study. The diagnostic value of RK in PWM was statistically significant. We speculated that the RK value may more sensitively reflect the limited radial diffusion of PWM.

With the same view as Vasung, the FA value of FWM has a certain diagnostic value in premature infants with brain development disorders (22). There was a significant difference in the FA value of FWM. It may be because FA is mainly affected by cell changes and fiber bundle density, while MK is majorly affected by the complexity of the microstructure (23, 24). Therefore, as a major functional area of the brain, we believe that FA value is sensitive in FWM.

This study confirmed that the correlations between MK, RK, and PMA were higher than the correlations between FA, MD, and PMA in the TH or CN. For homogeneous structures, such as gray matter, MK and RK are more sensitive as compared with FA and MD. MK and RK played an important role in detecting the development of isotropic tissues (such as gray matter) (25). In the gray matter region, the changes of MK and RK parameters may be related to the increased concentration of mature neuronal macromolecules and the decrease of tissue water content (26, 27) or to other special structures occurring in the development of gray matter. As an advanced and sensitive imaging technique, MK and RK can be used to detect the subtle structural changes of thalamic neurons in the premature infants. MK and RK can show hidden manifestations that FA and MD cannot detect *in vivo*. Therefore, we speculate that the MK and RK parameters have an advantage in reflecting the development of the deep nucleus in the premature infants. MK and RK parameters can better reflect the brain maturity of premature infants.

There were several limitations in this study. This study was only a cross-sectional study. Further verification of longitudinal data is still needed. In addition, the sample size was not large enough. Next, we will continue to enlarge the sample size. Finally, the PMA range of the study subjects was relatively large. If the same newborn brain template is used, the accuracy of image registration may be improved. The next step will be to establish brain templates for different segments of PMA.

## Conclusion

In conclusion, this study found that MK of ALIC and PLIC, RK of PWM and FA of FWM have some diagnostic value in detecting the brain developmental disorders in the premature infants. The MK and RK values of TH and CN can better reflect the brain maturity of the deep gray matter in the premature infants. These parameters could be used as reliable imaging markers for the diagnosis of the brain developmental disorders in the premature infants.

## Data Availability Statement

The original contributions presented in the study are included in the article/supplementary material, further inquiries can be directed to the corresponding author.

## Ethics Statement

The studies involving human participants were reviewed and approved by the Ethics Committee of the Third Affiliated Hospital of Zhengzhou University. Written informed consent to participate in this study was provided by the participants' legal guardian/next of kin.

## Author Contributions

XZ and CZ designed and wrote the research. BZ and JY performed the research and analyzed the data. KW modified language. ZZ collected data. XZ designed and funded the research. All authors contributed to the article and approved the submitted version.

## Funding

This research was funded by the National Natural Science Foundation of China, Grant No. 81870983; sponsor: XZ.

## Conflict of Interest

KW was employed by company GE Healthcare. The remaining authors declare that the research was conducted in the absence of any commercial or financial relationships that could be construed as a potential conflict of interest.

## Publisher's Note

All claims expressed in this article are solely those of the authors and do not necessarily represent those of their affiliated organizations, or those of the publisher, the editors and the reviewers. Any product that may be evaluated in this article, or claim that may be made by its manufacturer, is not guaranteed or endorsed by the publisher.

## References

[1] Ushida T Kidokoro H Nakamura N Katsuki S Imai K Nakano-Kobayashi T. Impact of maternal hypertensive disorders of pregnancy on brain volumes at term-equivalent age in preterm infants: a voxel-based morphometry study. Pregnancy Hypertens. (2021) 25:143–9. 10.1016/j.preghy.2021.06.003 34139669

[2] Baldoli C Scola E Della Rosa P Pontesilli S Longaretti R Poloniato A. Maturation of preterm newborn brains: a fMRI-DTI study of auditory processing of linguistic stimuli and white matter development. Brain Struct Funct. (2015) 220:3733–51. 10.1007/s00429-014-0887-5 25244942

[3] Hui E Cheung M Chan K Wu E. B-value dependence of DTI quantitation and sensitivity in detecting neural tissue changes. Neuroimage. (2010) 49:2366–74. 10.1016/j.neuroimage.2009.10.022 19837181

[4] Li X Gao J Wang M Zheng J Li Y Hui E. Characterization of extensive microstructural variations associated with punctate white matter lesions in preterm neonates. AJNR American journal of neuroradiology. (2017) 38:1228–34. 10.3174/ajnr.A5226 28450434 PMC7960104

[5] Fischi-Gómez E Vasung L Meskaldji D Lazeyras F Borradori-Tolsa C Hagmann P. Structural brain connectivity in school-age preterm infants provides evidence for impaired networks relevant for higher order cognitive skills and social cognition. Cerebral cortex. (2015) 25:2793–805. 10.1093/cercor/bhu073 24794920

[6] Palacios E Owen J Yuh E Wang M Vassar M Ferguson A. The evolution of white matter microstructural changes after mild traumatic brain injury: a longitudinal DTI and NODDI study. Sci Adv. (2020) 6:eaaz6892. 10.1126/sciadv.aaz6892 32821816 PMC7413733

[7] Lu H Jensen J Ramani A Helpern J. Three-dimensional characterization of non-gaussian water diffusion in humans using diffusion kurtosis imaging. NMR in biomedicine. (2006) 19:236–47. 10.1002/nbm.1020 16521095

[8] Guan J Ma X Geng Y Qi D Shen Y Shen Z. Diffusion Kurtosis Imaging for detection of early brain changes in Parkinson's disease. Front Neurol. (2019) 10:1285. 10.3389/fneur.2019.01285 31920913 PMC6914993

[9] Cheung M Hui E Chan K Helpern J Qi L Wu E. Does diffusion kurtosis imaging lead to better neural tissue characterization? A rodent brain maturation study. NeuroImage. (2009) 45:386–92. 10.1016/j.neuroimage.2008.12.018 19150655

[10] Paydar A Fieremans E Nwankwo J Lazar M Sheth H Adisetiyo V. Diffusional kurtosis imaging of the developing brain. AJNR Am J Neuroradiol. (2014) 35:808–14. 10.3174/ajnr.A3764 24231848 PMC7965814

[11] Shi J Yang S Wang J Huang S Yao Y Zhang S. Detecting normal pediatric brain development with diffusional kurtosis imaging. Eur J Radiol. (2019) 120:108690. 10.1016/j.ejrad.2019.108690 31605964

[12] Dibble M Ang J Mariga L Molloy E Bokde A. Diffusion tensor imaging in very preterm, moderate-late preterm and term-born neonates: a systematic review. J Pediatr. (2021). 10.1016/j.jpeds.2021.01.008 33453200

[13] Choi Y Lee J Lee J Lee J Lee Y Ahn J Lee H. Delayed maturation of the middle cerebellar peduncles at near-term age predicts abnormal neurodevelopment in preterm infants. Neonatology 2021:1-10. 10.1159/000512921 33503618 PMC8117383

[14] Rosch K Mostofsky S. Development of the frontal lobe. Handb Clin Neurol. (2019) 163:351–67. 10.1016/B978-0-12-804281-6.00019-7 31590741

[15] Grossman E Ge Y Jensen J Babb J Miles L Reaume J. Thalamus and cognitive impairment in mild traumatic brain injury: a diffusional kurtosis imaging study. J Neurotrauma. (2012) 29:2318–27. 10.1089/neu.2011.1763 21639753 PMC3430483

[16] Mukherjee P Miller J Shimony J Philip J Nehra D Snyder A. Diffusion-tensor MR imaging of gray and white matter development during normal human brain maturation. AJNR American journal of neuroradiology. (2002) 23:1445–56. 12372731 PMC7976805

[17] Mukherjee P Miller J Shimony J Conturo T Lee B Almli C. Normal brain maturation during childhood: developmental trends characterized with diffusion-tensor MR imaging. Radiology. (2001) 221:349–58. 10.1148/radiol.2212001702 11687675

[18] Ling X Tang W Liu G Huang L Li B Li X. Assessment of brain maturation in the preterm infants using diffusion tensor imaging (DTI) and enhanced T2 star weighted angiography (ESWAN). Eur J Radiol. (2013) 82:e476–483. 10.1016/j.ejrad.2013.04.003 23639775

[19] Dubois J Dehaene-Lambertz G Perrin M Mangin J Cointepas Y Duchesnay E. Asynchrony of the early maturation of white matter bundles in healthy infants: quantitative landmarks revealed noninvasively by diffusion tensor imaging. Hum Brain Mapp. (2008) 29:14–27. 10.1002/hbm.20363 17318834 PMC6870818

[20] Olivieri B Rampakakis E Gilbert G Fezoua A Wintermark P. Myelination may be impaired in neonates following birth asphyxia. NeuroImage Clinical. (2021) 31:102678. 10.1016/j.nicl.2021.102678 34082365 PMC8182124

[21] Yoshida S Oishi K Faria A Mori S. Diffusion tensor imaging of normal brain development. Pediatric radiology. (2013) 43:15–27. 10.1007/s00247-012-2496-x 23288475 PMC3703661

[22] Vasung L Fischi-Gomez E Hüppi P. Multimodality evaluation of the pediatric brain: DTI and its competitors. Pediatr Radiol. (2013) 43:60–8. 10.1007/s00247-012-2515-y 23288478

[23] Ouyang M Jeon T Sotiras A Peng Q Mishra V Halovanic C. Differential cortical microstructural maturation in the preterm human brain with diffusion kurtosis and tensor imaging. Proc Natl Acad Sci USA. (2019) 116:4681–8. 10.1073/pnas.1812156116 30782802 PMC6410816

[24] Nossin-Manor R Card D Raybaud C Taylor M Sled J. Cerebral maturation in the early preterm period-A magnetization transfer and diffusion tensor imaging study using voxel-based analysis. Neuroimage. (2015) 112:30–42. 10.1016/j.neuroimage.2015.02.051 25731990

[25] McKenna F Miles L Donaldson J Castellanos F Lazar M. Diffusion kurtosis imaging of gray matter in young adults with autism spectrum disorder. Sci Rep. (2020) 10:21465. 10.1038/s41598-020-78486-w 33293640 PMC7722927

[26] Das S Wang J Bing L Bhetuwal A Yang H. Regional values of diffusional kurtosis estimates in the healthy brain during normal aging. Clin Neuroradiol. (2017) 27:283–98. 10.1007/s00062-015-0490-z 26729366

[27] Gullion L Stansell J Moss H Jenkins D Aljuhani T Coker-Bolt P. The impact of early neuroimaging and developmental assessment in a preterm infant diagnosed with cerebral palsy. Case Rep Pediatr. (2019) 2019:9612507. 10.1155/2019/9612507 30881719 PMC6383416

